# Developing a Reporting Guideline for Social and Psychological Intervention Trials

**DOI:** 10.1177/1049731513498118

**Published:** 2013-11

**Authors:** Sean Grant, Paul Montgomery, Sally Hopewell, Geraldine Macdonald, David Moher, Evan Mayo-Wilson

**Affiliations:** 1 Centre for Evidence-Based Intervention, University of Oxford, Oxford, UK; 2 Centre for Statistics in Medicine, University of Oxford, Oxford, UK; 3 Institute of Child Care Research, Queen’s University Belfast, Belfast, UK; 4 Clinical Epidemiology Program, Ottawa Hospital Research Institute, Centre for Practice-Changing Research (CPCR), The Ottawa Hospital, Ottawa, ON, Canada; 5 Research Department of Clinical, Educational & Health Psychology, Centre for Outcomes Research and Effectiveness, University College London, London, UK

**Keywords:** randomized controlled trial, RCT, CONSORT-SPI, reporting guideline, reporting standards

## Abstract

Social and psychological interventions are often complex. Understanding randomized controlled trials (RCTs) of these complex interventions requires a detailed description of the interventions tested and the methods used to evaluate them; however, RCT reports often omit, or inadequately report, this information. Incomplete and inaccurate reporting hinders the optimal use of research, wastes resources, and fails to meet ethical obligations to research participants and consumers. In this article, we explain how reporting guidelines have improved the quality of reports in medicine and describe the ongoing development of a new reporting guideline for RCTs: Consolidated Standards of Reporting Trials-SPI (an extension for social and psychological interventions). We invite readers to participate in the project by visiting our website, in order to help us reach the best-informed consensus on these guidelines (http://tinyurl.com/CONSORT-study).

## Introduction

Social and psychological interventions aim to improve physical health, mental health, and associated social outcomes. They are often complex and typically involve multiple, interacting intervention components (e.g., several behavior change techniques) that may act and target outcomes on several levels (e.g., individual, family, and community; Medical Research Council [MRC], 2008). Moreover, these interventions may be contextually dependent upon the hard-to-control environments in which they are delivered (e.g., health care settings and correctional facilities; Bonell, 2002; Pawson, Greenhalgh, Harvey, & Walshe, 2004). The functions and processes of these interventions may be designed to accommodate particular individuals or contexts, taking on different forms while still aiming to achieve the same objective (Bonell, Fletcher, Morton, Lorenc, & Moore, 2012; Hawe, Shiell, & Riley, 2004).

Complex interventions are common in public health, psychology, education, social work, criminology, and related disciplines. For example, multisystemic therapy (MST) is an intensive intervention for juvenile offenders. Based on social ecological and family system theories, MST providers target a variety of individual, family, school, peer, neighborhood, and community influences on psychosocial and behavioral problems (Henggeler, Schoenwald, Rowland, & Cunningham, 2002). Treatment teams of professional therapists and caseworkers work with individuals, their families, and their peer groups to provide tailored services (Littell, Campbell, Green, & Toews, 2009). These services may be delivered in homes, social care, and community settings. Other examples of social and psychological interventions may be found in reviews by the Cochrane Collaboration (2013; e.g., the Developmental, Psychosocial, and Learning Problems Group; the Cochrane Public Health Group) and the Campbell Collaboration (2013).

To understand their effects and to keep services up to date, academics, policy makers, journalists, clinicians, and consumers rely on research reports of intervention studies in scientific journals. Such reports should explain the methods, including the design, delivery, uptake, and context of interventions, as well as subsequent results. Accurate, complete, and transparent reporting is essential for readers to make best use of new evidence, to achieve returns on research investment, to meet ethical obligations to research participants and consumers of interventions, and to minimize waste in research. However, reports of randomized controlled trials (RCTs) are often poorly reported within and across disciplines including criminology (Perry, Weisburd, & Hewitt, 2010), social work (Naleppa & Cagle, 2010), education (Torgerson, Torgerson, Birks, & Porthouse, 2005), psychology (Michie et al., 2011; Stinson, McGrath, & Yamada, 2003), and public health (Semaan et al., 2002). Biomedical researchers have developed guidelines to improve the reporting of RCTs of health-related interventions (Schulz, Altman, & Moher, for the CONSORT Group, 2010). However, many social and behavioral scientists have not fully adopted these guidelines, which may not be wholly adequate for social and psychological interventions in their current form (Bonell, Oakley, Hargreaves, Strange, & Rees, 2006; Davidson et al., 2003; Perry et al., 2010; Stinson et al., 2003). Because of the unique features of these interventions, updated reporting guidance is needed.

This article describes the development of a reporting guideline that aims to improve the quality of reports of RCTs of social and psychological interventions. We explain how reporting guidelines have improved the quality of reports in medicine, and why guidelines have not yet improved the quality of reports in other disciplines. We then introduce a plan to develop a new reporting guideline for RCTs—Consolidated Standards of Reporting Trials (CONSORT)-SPI (an extension for social and psychological interventions)—which will be written using recommend techniques for guideline development and dissemination (Moher, Schulz, Simera, & Altman, 2010). Wide stakeholder involvement and consensus are needed to create a useful, acceptable, and evidence-based guideline, so we hope to recruit stakeholders from multiple disciplines and professions.

Randomized trials are not the only rigorous method for evaluating interventions; many alternatives exist when RCTs are not possible or appropriate due to scientific, practical, and ethical concerns (Bonell et al., 2011). Nonetheless, RCTs are important to policy makers, practitioners, scientists, and service users, as they are generally considered the most valid and reliable research method for estimating the effectiveness of interventions (Chalmers, 2003). Moreover, many of the issues faced in reporting RCTs also relate to other evaluation designs. As a result, this project will focus on standards for RCTs, which could then also inform the development of future guidelines for other evaluation designs.

### Impact of CONSORT Guidelines

Reporting guidelines list (in the form of a checklist) the minimum information required to understand the methods and results of studies. They do not prescribe research conduct, but facilitate the writing of transparent reports by authors and appraisal of reports by research consumers. For example, the CONSORT Statement 2010 is an evidence-based guideline; to identify items, the developers reviewed evidence of trial design and conduct that could contribute to bias. Using consensus methods, they developed a checklist of 25 items and a flow diagram (Schulz et al., 2010). CONSORT has improved the reporting of thousands of medical experiments (Turner et al., 2012). It has been endorsed by over 600 journals (Moher, Altman, Schulz, & Elbourne, 2004), and it is supported by the Institute of Educational Sciences (Torgerson et al., 2005). CONSORT is the only guideline for reporting RCTs that has been developed with such rigor, and it has remained more prominent that any other guideline for over 15 years; for greatest impact, any further reporting guidelines related to RCTs should be developed in collaboration with the CONSORT Group.

### Limitations of Previous Reporting Guidelines for Social and Psychological Interventions

Researchers and journal editors in the social and behavioral sciences are generally aware of CONSORT but often object that it is not fully appropriate for social and psychological interventions (Bonell et al., 2006; Davidson et al., 2003; Perry et al., 2010; Stinson et al., 2003). As a result, uptake of CONSORT guidelines in these disciplines is low. While some criticisms are due to inaccurate perceptions about common features of RCTs across disciplines, many relate to real limitations for social and psychological interventions (Mayo-Wilson, 2007). For example, CONSORT is most relevant to RCTs in medical disciplines; it was developed by biostatisticians and medical researchers with minimal input from experts in other disciplines. Journal editors, as well as social and behavioral science researchers, believe there is a need to include appropriate stakeholders in developing a new, targeted guideline to improve uptake in their disciplines (Gill, 2011; Torgerson et al., 2005). The CONSORT Group has produced *extensions* of the original CONSORT Statement relevant to social and psychological interventions, such as additional checklists for cluster (Campbell, Elbourne, & Altman, 2004), nonpharmacological (Boutron et al., 2008a), pragmatic (Zwarenstein et al., 2008), and quality of life RCTs (Calvert, Blazeby, Revicki, Moher, & Brundage, 2011). These extensions provide important insights, but complex social and psychological interventions, for example, include multiple, interacting components at several levels, with various outcomes. These RCTs require use of several extensions at once, creating a barrier to guideline uptake; increasing intervention complexity also gives rise to new issues that are not included in existing guidelines. Therefore, simply disseminating CONSORT guidelines as they stand is insufficient, as this would not address the need for editors and authors to “buy-in” to this process. To improve uptake in these disciplines, CONSORT guidelines need to be extended to specifically address the important features of social and psychological interventions.

Social and behavioral scientists have developed other reporting guidelines, including the Workgroup for Intervention Development and Evaluation Research (WIDER) Recommendations for behavioral change interventions (Abraham, for the WIDER, 2009; Michie et al., 2011), the American Educational Research Association’s (AERA, 2006) Standards for Reporting Research, the REPOrting of Studies in Education (REPOSE) guidelines for primary research in education (Newman & Elbourne, 2004), and the Journal Article Reporting Standards (JARS) of the American Psychological Association (APA) Publications and Communications Board Working Group on JARS (2008). While they address issues not covered by the CONSORT Statement and its extensions, these guidelines (except for JARS; APA Publications and Communications Board Working Group on JARS, 2008) do not provide specific guidance for RCTs. Moreover, compared with the CONSORT Statement and its official extensions, guidelines in the social and behavioral sciences have not consistently followed optimal techniques for guideline development and dissemination that are recommended by international leaders in the advancement of reporting guidelines (Moher, Schulz, et al., 2010), such as the use of systematic literature reviews and formal consensus methods to select reporting standards (Grant, Montgomery, & Mayo-Wilson, 2012). Researchers in public health, psychology, education, social work, and criminology have noted that these guidelines could be more “user-friendly,” and dissemination could benefit from up-to-date knowledge transfer techniques (Abraham, 2009; Armstrong et al., 2008; Davidson et al., 2003; Naleppa & Cagle, 2010; Perry & Johnson, 2008; Stinson et al., 2003; Torgerson et al., 2005).

For example, JARS—a notable and valuable guideline for empirical psychological research—is endorsed by few journals outside of the APA, whereas CONSORT is endorsed by hundreds of journals internationally. According to ISI Web of Knowledge and Google Scholar citations, JARS is cited approximately a dozen times annually, while CONSORT guidelines are cited hundreds of times per year. Moreover, the APA commissioned a select group of APA journal editors and reviewers to develop JARS, and the group based most of their work on existent CONSORT guidelines; by comparison, official CONSORT extensions have been developed using rigorous consensus methods, have involved various international stakeholders in guideline development and dissemination, and update content on the most recent scientific literature. Nonetheless, no current CONSORT guideline adequately addresses the unique features of social and psychological interventions. This new CONSORT extension will incorporate lessons from previous extensions, reporting guidelines, and the research literature to aid the critical appraisal, replication, and uptake of this research.

### Aspects of Internal Validity

Internal validity is the extent to which the results of a study may be influenced by bias. Like other study designs, the validity of RCTs depends on high-quality execution. Poorly conducted RCTs can produce more biased results than well-conducted RCTs and well-conducted nonrandomized studies (Pildal et al., 2007; Prescott et al., 1999). For example, evidence indicates that RCTs that do not adequately conceal the randomization sequence can exaggerate effect estimates by up to 30% (Schulz, Chalmers, Hayes, & Altman, 1995), while low-quality reports of these RCTs are associated with effect estimates exaggerated by up to 35% (Moher et al., 1999). Social and psychological intervention RCTs are susceptible to these risks of bias as well.

Some aspects of internal validity, although included in CONSORT, remain poorly reported—even in the least complex social and psychological intervention studies. Reports of RCTs should describe procedures for minimizing selection bias, but reports often omit information about random sequence generation and allocation concealment (Ladd, McCrady, Manuel, & Campbell, 2010; Perry & Johnson, 2008), and psychological journals report methods of sequence generation less frequently than medical journals (Stinson et al., 2003). A review of educational reports found no studies that adequately reported allocation concealment (Torgerson et al., 2005), and reports in criminology often lack information about randomization procedures (Gill, 2011; Perry et al., 2010). RCTs of social and psychological interventions may also use nontraditional randomization techniques, such as stepped wedge or natural allocation (MRC, 2011), which need to be thoroughly described. In addition, reports of social and psychological intervention trials often fail to include details about trial registration, protocols, and adverse events (Ladd et al., 2010; Perry & Johnson, 2008), which may include important negative consequences at individual, familial, and community levels.

Other aspects of CONSORT may require greater emphasis or modification for RCTs of social and psychological interventions. In developing this CONSORT extension, we expect to identify new items and to adapt existing items that relate to the internal validity. These may include items discussed during the development of previous CONSORT extensions or other guidelines, as well as items suggested by participants in this project. For example, it may not be possible to blind participants and providers of interventions, but blinding of outcome assessors is often possible but rarely reported, and few studies explain if blinding was maintained or how lack of blinding was handled (Davidson et al., 2003; Ladd et al., 2010; Perry & Johnson, 2008). In social and psychological intervention studies, outcome measures are often subjective, variables may relate to latent constructs, and information may come from multiple sources (e.g., participants and providers). While an issue in other areas of research, the influence on RCT results of the quality of subjective outcome measures in social and psychological intervention research has long been highlighted, given their prevalence in social and psychological intervention research (Marshall et al., 2000). Descriptions of the validity, reliability, and psychometric properties of such measures are therefore particularly useful for social and psychological intervention trials, especially when they are not widely available or discussed in the research literature (Campbell et al., 2004; Fraser, Galinsky, Richman, & Day, 2009). Moreover, multiple measures may be analyzed in several ways, so authors need to transparently report which procedures were performed and to explain their rationale.

### Aspects of External Validity

External validity is the extent to which a study’s results are applicable in other settings or populations. Currently, given that RCTs are primarily designed to increase the internal validity of study findings, the CONSORT Statement gives relatively little attention to external validity. While high internal validity is an important precondition for any discussion of an RCT’s external validity, updating the CONSORT Statement to include more information about external validity is critical for the relevance and uptake of a CONSORT extension for social and psychological interventions. These interventions may be influenced by context, as different underlying social, institutional, psychological, and physical structures may yield different causal and probabilistic relations between interventions and observed outcomes. Contextual information is necessary to compare the effectiveness of an intervention across time and place (Cartwright & Munro, 2010). Lack of information relevant to external validity may prevent practitioners or policy makers from using evidence appropriately to inform decision making; yet, existing guidelines do not adequately explain how authors should describe (a) how interventions work, (b) for whom, and (c) under what conditions (Moore & Moore, 2011).

First, it is useful for authors to explain the key components of interventions, how those components could be delivered, and how they relate to the outcomes selected. At present, authors can follow current standards for reporting interventions without providing adequate details about complex interventions (Shepperd et al., 2009). Many reports neither contain sufficient information about the interventions tested nor reference treatment manuals (Glasziou, Meats, Heneghan, & Shepperd, 2008). Providing logic models—as described in the MRC Framework for Complex Interventions (Craig et al., 2008)—or presenting theories of change can help elucidate links in causal chains that can be tested, identify important mediators and moderators, and facilitate syntheses in reviews (Ivers et al., 2012). Moreover, interventions are rarely implemented exactly as designed, and complex interventions may be designed to be implemented with some flexibility, in order to accommodate differences across participants (Hawe et al., 2004), so it is important to report how interventions were *actually delivered* by providers and *actually received* by participants (Hardeman et al., 2008). Particularly for social and psychological interventions, the integrity of implementing the intended functions and processes of the intervention are essential to understand (Hawe et al., 2004). As RCTs of a particular intervention can yield different relative effects depending on the nature of the control groups, information about delivery and uptake should be provided for *all* trial arms (McGrath, Stinson, & Davidson, 2003).

Second, reports should describe recruitment processes and representativeness of samples. Participants in RCTs of social and psychological intervention are often recruited outside of routine practice settings via processes that differ from routine services (AERA, 2006). An intervention that works for one group of people may not work for people living in different cultures or physical spaces, or it may not work for people with slightly different problems and comorbidities. Enrolling in an RCT can be a complex process that affects the measured and unmeasured characteristics of participants, and recruitment may differ from how users normally access interventions. Well-described RCT reports will include the characteristics of all participants (volunteers, those who enrolled, and those who completed) in sufficient detail for readers to assess the comparability of the study sample to populations and in everyday services (AERA, 2006; APA Publications and Communications Board Working Group on JARS, 2008; Evans & Brown, 2003)

Finally, given that these interventions often occur in social environments, reports should describe factors of the RCT context that are believed to support, attenuate, or frustrate observed effects (Moore, 2002). Interventions may differ across groups of different social or socioeconomic positions, and equity considerations should be addressed explicitly (Tugwell et al., 2010; Welch et al., 2012). Several aspects of setting and implementation may be important to consider, such as administrative support, staff training and supervision, organizational resources, the wider service system, and concurrent political or social events (Bonell et al., 2012; Fixsen, Naoom, Blase, Friedman, & Wallace, 2005; Shepperd et al., 2009; Wang, Moss, & Hiller, 2006). Reporting process evaluations may help understand mechanisms and outcomes.

### Developing a New CONSORT Extension

This new reporting guideline for RCTs of social and psychological interventions will be an official extension of the CONSORT Statement. Optimally, it will help improve the reporting of these studies. Like other official CONSORT extensions (Boutron et al., 2008a; Campbell et al., 2004; Hopewell et al., 2008; Zwarenstein et al., 2008), this guideline will be integrated with the CONSORT Statement and previous extensions, and updates of the CONSORT Statement may incorporate references to this extension.

The project is being led by an international collaboration of researchers, methodologists, guideline developers, funders, service providers, journal editors, and consumer advocacy groups. We will be recruiting participants in a manner similar to other reporting guideline initiatives—identifying stakeholders through literature reviews, the project’s International Advisory Group, and stakeholder-initiated interest in the project (Michie et al., 2011; Schulz et al., 2010). We hope to recruit stakeholders with expertise from all related disciplines and regions of the world, including low- and middle-income countries. Methodologists will identify items that relate to known sources of bias, and they will identify items that facilitate systematic reviews and research synthesis. Funders will consider how the guideline can aid the assessment of grant applications for RCTs and methodological innovations in intervention evaluation. Practitioners will identify information that can aid decision making. Journal editors will identify practical steps to implement the guideline and to ensure uptake.

We will use consensus techniques to reduce bias in group decision making and to promote widespread guideline uptake and knowledge translation activities upon project completion (Murphy et al., 1998). Following rigorous reviews of existing guidelines and current reporting quality, we will conduct an online Delphi process to identify a prioritized list of reporting items to consider for the extension. That is, we will invite a group of experts to electronically answer questions about reporting items and to suggest further questions. We will circulate their feedback to the group and ask a second round of questions. The Delphi process will capture a variety of international perspectives and allow participants to share their views anonymously. Following the Delphi process, we will host a consensus meeting to review the findings and to generate a list of minimal reporting standards, mirroring the development of previous CONSORT guidelines (Boutron et al., 2008b; Schulz et al., 2010; Zwarenstein et al., 2008).

Together, participants in this process will create a checklist of reporting items and a flowchart for reporting social and psychological intervention RCTs. In addition, we will develop an Explanation and Elaboration (E&E) document to explain the scientific rationale for each recommendation and to provide examples of clear reporting; a similar document was developed by the CONSORT group to help disseminate a better understanding for each included checklist item (Moher, Hopewell, et al., 2010). This document will help persuade editors, authors, and funders of the importance of the guideline. It will be a useful pedagogical tool, helping students and researchers understand the methods for conducting RCTs of social and psychological interventions, and it will help authors meet the guideline requirements (Moher, Schulz, et al., 2010).

The success of this project depends on widespread involvement and agreement among key international stakeholders in research, policy, and practice. For example, previous developers have obtained guideline endorsement by journal editors who require authors and peer reviewers to use the guideline during article submission and who must enforce journal article word limits (Michie, Fixsen, Grimshaw, & Eccles, 2009). Many journal editors have already agreed to participate, and we hope other researchers and stakeholders will volunteer their time and expertise.

## Conclusion

Reporting guidelines help us use scarce resources efficiently and ethically. RCTs are expensive, and the public have a right to expect returns on their investments through transparent, usable reports. When RCT reports cannot be used (for whatever reason), resources are wasted. Participants contribute their time and put themselves at risk of harm to generate evidence that will help others, and researchers should disseminate that information effectively (Davidson et al., 2003). Policy makers benefit from research when developing effective, affordable standards of practice and choosing which programs and services to fund. Administrators and managers are required to make contextually appropriate decisions. Transparent reporting of primary studies is essential for their inclusion in systematic reviews that inform these activities. For example, there is the need to determine if primary studies are comparable, examine biases within included studies, assess the generalizability of results, and implement effective interventions. Finally, we hope this guideline will reduce the effort and time required for authors to write reports of RCTs.

RCTs are not the only valid method for evaluating interventions (Bonell et al., 2011) nor are they the only type of research that would benefit from better reporting (Goldbeck & Vitiello, 2011). Colleagues have identified the importance of reporting standards for other types of research, including observational (von Elm et al., 2007), quasi-experimental (Des Jarlais, Lyles, Crepaz, & the TREND Group, 2004), and qualitative studies (Tong, Sainsbury, & Craig, 2007). This guideline is the first step toward improving reports of many designs for evaluating social and psychological interventions, which we hope will be addressed by this and future projects. We invite stakeholders from disciplines that frequently research these interventions to join this important effort and participate in guideline development by visiting our website, where they can find more information about the project, updates on its progress, and sign up to be involved (http://tinyurl.com/CONSORT-study).

## References

[bibr1-1049731513498118] Abraham C., for the Workgroup for Intervention Development and Evaluation Research. (2009). WIDER recommendations to improve reporting of the content of behaviour change interventions. Retrieved November 14, 2012, from http://interventiondesign.co.uk/wp-content/uploads/2009/02/wider-recommendations.pdf

[bibr2-1049731513498118] American Educational Research Association. (2006). Standards for reporting on empirical social science research in AERA publications. Educational Researcher, 35, 33–40

[bibr3-1049731513498118] American Psychological Association Publications and Communications Board Working Group on Journal Article Reporting Standards. (2008). Reporting standards for research in psychology: Why do we need them? what might they be? American Psychologist, 63, 839–851 19086746 10.1037/0003-066X.63.9.839 PMC2957094

[bibr4-1049731513498118] Armstrong R. Waters E. Moore L. Riggs E. Cuervo L. G. Lumbiganon P. Hawe P. (2008). Improving the reporting of public health intervention research: Advancing TREND and CONSORT. Journal of Public Health, 30, 103–109 18204086 10.1093/pubmed/fdm082

[bibr5-1049731513498118] Bonell C. (2002). The utility of randomized controlled trials of social interventions: An examination of two trials of HIV prevention. Critical Public Health, 12, 321–334

[bibr6-1049731513498118] Bonell C. Fletcher A. Morton M. Lorenc T. Moore L. (2012). Realist randomised controlled trials: A new approach to evaluating complex public health interventions. Social Science & Medicine, 75, 2299–2306 22989491 10.1016/j.socscimed.2012.08.032

[bibr7-1049731513498118] Bonell C. Oakley A. Hargreaves J. Strange V. Rees R. (2006). Assessment of generalisability in trials of health interventions: Suggested framework and systematic review. British Medical Journal, 333, 346–349 16902217 10.1136/bmj.333.7563.346 PMC1539056

[bibr8-1049731513498118] Bonell C. P. Hargreaves J. Cousens S. Ross D. Hayes R. Petticrew M. Kirkwood B. R. (2011). Alternatives to randomisation in the evaluation of public health interventions: Design challenges and solutions. Journal of Epidemiological Community Health, 65, 582–587 10.1136/jech.2008.082602 19213758

[bibr9-1049731513498118] Boutron I. Moher D. Altman D. G. Schulz K. Ravaud P. for the CONSORT group. (2008a). Extending the CONSORT statement to randomized trials of nonpharmacologic treatment: Explanation and Elaboration. Annals of Internal Medicine, 148, 295–309 18283207 10.7326/0003-4819-148-4-200802190-00008

[bibr10-1049731513498118] Boutron I. Moher D. Altman D. G. Schulz K. Ravaud P. , for the CONSORT group. (2008b). Methods and processes of the CONSORT group: Example of an extension for trials assessing nonpharmacologic treatments. Annals of Internal Medicine, 148, W60–W67 18283201 10.7326/0003-4819-148-4-200802190-00008-w1

[bibr11-1049731513498118] Calvert M. Blazeby J. Revicki D. Moher D. Brundage M. (2011). Reporting quality of life in clinical trials: A CONSORT extension. The Lancet, 378, 1684–1685 10.1016/S0140-6736(11)61256-7 22078674

[bibr12-1049731513498118] Campbell Collaboration. (2013). Retrieved April 26, 2013, from http://campbellcollaboration.org/

[bibr13-1049731513498118] Campbell M. K. Elbourne D. R. Altman D. G. (2004). CONSORT statement: Extension to cluster randomised trials. British Medical Journal, 328, 702–708 15031246 10.1136/bmj.328.7441.702 PMC381234

[bibr14-1049731513498118] Cartwright N. Munro E. (2010). The limitations of randomized controlled trials in predicting effectiveness. Journal of Evaluation in Clinical Practice, 16, 260–266 20367845 10.1111/j.1365-2753.2010.01382.x

[bibr15-1049731513498118] Chalmers I. (2003). Trying to do more good than harm in policy and practice: The role of rigorous, transparent, up-to-date evaluations. The ANNALS of the American Academy of Political and Social Science, 589, 22–40

[bibr16-1049731513498118] Cochrane Collaboration. (2013). Retrieved April 26, 2013, from http://www.cochrane.org/

[bibr17-1049731513498118] Craig P. Dieppe P. Macintyre S. Mitchie S. Nazareth I. Petticrew M. (2008). Developing and evaluating complex interventions: The new Medical Research Council guidance. British Medical Journal, 337, 979–983 10.1136/bmj.a1655 PMC2769032 18824488

[bibr18-1049731513498118] Davidson K. W. Goldstein M. Kaplan R. M. Kaufmann P. G. Knatterud G. L. Orleans C. T. Spring B.…Whitlock E. P (2003). Evidence-based behavioural medicine: What is it and how do we achieve it? Annals of Behavioral Medicine, 26, 161–171 14644692 10.1207/S15324796ABM2603_01

[bibr19-1049731513498118] Des Jarlais D. C. Lyles C. Crepaz N. , & the TREND Group. (2004). Improving the reporting quality of nonrandomized evaluations of behavioral and public health interventions: The TREND statement. American Journal of Public Health, 94, 361–366 14998794 10.2105/ajph.94.3.361 PMC1448256

[bibr20-1049731513498118] Evans T. Brown H. (2003). Road traffic crashes: Operationalizing equity in the context of health sector reform. Injury Control and Safety Promotion, 10, 11–12 12772480 10.1076/icsp.10.1.11.14117

[bibr21-1049731513498118] Fixsen D. L. Naoom S. F. Blase K. A. Friedman R. M. Wallace F. (2005). Implementation research: A synthesis of the literature. Tampa, FL: University of South Florida

[bibr22-1049731513498118] Fraser M. W. Galinsky M. J. Richman J. M. Day S. H. (2009). Intervention research: Developing social programs. Oxford, England: Oxford University Press

[bibr23-1049731513498118] Gill C. E. (2011). Missing links: How descriptive validity impacts the policy relevance of randomized controlled trials in criminology. Journal of Experimental Criminology, 7, 201–224

[bibr24-1049731513498118] Glasziou P. Meats E. Heneghan C. Shepperd S. (2008). What is missing from descriptions of treatment in trials and reviews? British Medical Journal, 336, 1472–1474 18583680 10.1136/bmj.39590.732037.47 PMC2440840

[bibr25-1049731513498118] Goldbeck L. Vitiello B. (2011). Reporting clinical trials of psychosocial interventions in child and adolescent psychiatry and mental health. Child and Adolescent Psychiatry and Mental Health, 5, 4 21356036 10.1186/1753-2000-5-4 PMC3056804

[bibr26-1049731513498118] Grant S. Montgomery P. Mayo-Wilson E. (2012). Development of a CONSORT extension for interventions in public health and related disciplines. The Lancet, 380, S14 Retrieved April 26, 2013, from http://www.thelancet.com/abstracts/public-health-science-in-the-uk

[bibr27-1049731513498118] Hardeman W. Michie S. Fanshawe T. Prevost A. T. McLoughlin K. Kinmonth A. L. (2008). Fidelity of delivery of a physical activity intervention: Predictors and consequences. Psychology & Health, 23, 11–24 10.1080/08870440701615948 25159904

[bibr28-1049731513498118] Hawe P. Shiell A. Riley T. (2004). Complex interventions: How “out of control” can a randomised controlled trial be? British Medical Journal, 328, 1561–1563 15217878 10.1136/bmj.328.7455.1561 PMC437159

[bibr29-1049731513498118] Henggeler S. W. Schoenwald S. K. Rowland M. D. Cunningham P. B. (2002). Serious emotional disturbances in children and adolescents: Multisystemic therapy. New York, NY: Guilford

[bibr30-1049731513498118] Hopewell S. Clarke M. Moher D. Wager E. Middleton P. Altman D. G. Schulz K. F., & the CONSORT Group. (2008). CONSORT for reporting randomised trials in journal and conference abstracts. Lancet, 371, 281–283 18221781 10.1016/S0140-6736(07)61835-2

[bibr31-1049731513498118] Ivers N. Jamtvedt G. Flottorp S. Young J. M. Odgaard-Jensen J. French S. D.…Oxman A. D. (2012). Audit and feedback: Effects on professional practice and healthcare outcomes. Cochrane Database of Systematic Reviews, 6, CD000259 22696318 10.1002/14651858.CD000259.pub3 PMC11338587

[bibr32-1049731513498118] Ladd B. O. McCrady B. S. Manuel J. K. Campbell W. (2010). Improving the quality of reporting alcohol outcome studies: Effects of the CONSORT statement. Addictive Behaviors, 35, 660–666 20207490 10.1016/j.addbeh.2010.02.009

[bibr33-1049731513498118] Littell J. H. Campbell M. Green S. Toews B. (2009). Multisystemic therapy for social, emotional, and behavioral problems in youth aged 10-17. Cochrane Library of Systematic Reviews, 4, CD004797

[bibr34-1049731513498118] Marshall M. Lockwood A. Bradley C. Adams C. Joy C. Fenton M. (2000). Unpublished rating scales: A major source of bias in randomised controlled trials of treatments for schizophrenia. British Journal of Psychiatry, 176, 249–252 10755072 10.1192/bjp.176.3.249

[bibr35-1049731513498118] Mayo-Wilson E. (2007). Reporting implementation in randomized trials: Proposed additions to the consolidated standards of reporting trials statement. American Journal of Public Health, 97, 630–633 17329641 10.2105/AJPH.2006.094169 PMC1829360

[bibr36-1049731513498118] McGrath P. J. Stinson J. Davidson K. (2003). Commentary: The journal of pediatric psychology should adopt the CONSORT statement as a way of improving the evidence base in pediatric psychology. Journal of Pediatric Psychology, 28, 169–171 12654940 10.1093/jpepsy/jsg002

[bibr37-1049731513498118] Medical Research Council. (2008). A framework for development and evaluation of RCTs for complex interventions to improve health. London, England: Author

[bibr38-1049731513498118] Medical Research Council. (2011). Using natural experiments to evaluate population health interventions: Guidance for producers and users of evidence. London, England: Author

[bibr39-1049731513498118] Michie S. Abraham C. Eccles M. P. Francis J. J. Hardeman W. Johnston M. (2011). Strengthening evaluation and implementation by specifying components of behaviour change interventions: A study protocol. Implementation Science, 6, 10 21299860 10.1186/1748-5908-6-10 PMC3041694

[bibr40-1049731513498118] Michie S. Fixsen D. Grimshaw J. M. Eccles M. P. (2009). Specifying and reporting complex behaviour change interventions: The need for a scientific method. Implementation Science, 4, 40 19607700 10.1186/1748-5908-4-40 PMC2717906

[bibr41-1049731513498118] Moher D. Altman D. G. Schulz K. F. Elbourne D. R. (2004). Opportunities and challenges for improving the quality of reporting clinical research: CONSORT and beyond. Canadian Medical Association Journal, 171, 349–350 15313995 10.1503/cmaj.1040031 PMC509049

[bibr42-1049731513498118] Moher D. Hopewell S. Schultz K. F. Montori V. Gøtzsche P. C. Devereaux P. J.…Altman D. G. (2010). CONSORT 2010 Explanation and Elaboration: Updated guidelines for reporting parallel group randomised trials. British Medical Journal, 340, c869 20332511 10.1136/bmj.c869 PMC2844943

[bibr43-1049731513498118] Moher D. Pham B. Jones A. Cook D. J. Jadad A. R. Moher M.…Klassen T. P. (1999). Does quality of reports of randomized trials affect estimates of intervention efficacy reported in meta-analyses? Lancet, 352, 609–613 9746022 10.1016/S0140-6736(98)01085-X

[bibr44-1049731513498118] Moher D. Schulz K. F. Simera I. Altman D. G. (2010). Guidance for developers of health research reporting guidelines. PLoS Medicine, 7, e1000217 10.1371/journal.pmed.1000217 PMC2821895 20169112

[bibr45-1049731513498118] Moore L. (2002). Research design for the rigorous evaluation of complex educational interventions: Lessons from health services research. Building Research Capacity, 1, 4–5

[bibr46-1049731513498118] Moore L. Moore G. F. (2011). Public health evaluation: Which designs work, for whom and under what circumstances? Journal of Epidemiology & Community Health, 65, 596–597 21665866 10.1136/jech.2009.093211

[bibr47-1049731513498118] Murphy M. K. Black N. A. Lamping D. L. McKee C. M. Sanders C. F. B. Askham J. Marteau T. (1998). Consensus development methods, and their use in clinical guideline development. Health Technology Assessment, 2, 1–88 9561895

[bibr48-1049731513498118] Naleppa M. J. Cagle J. G. (2010). Treatment fidelity in social work intervention research: A review of published studies. Research on Social Work Practice, 20, 674–681

[bibr49-1049731513498118] Newman M. Elbourne D. (2004). Improving the usability of educational research: Guidelines for the REPOrting of primary empirical research Studies in Education (The REPOSE Guidelines). Evaluation & Research in Education, 18, 201–212

[bibr50-1049731513498118] Pawson R. Greenhalgh T. Harvey G. Walshe K. (2004). Realist synthesis: An introduction. Manchester, England: ESRC Research Methods Programme, University of Manchester

[bibr51-1049731513498118] Perry A. E. Johnson M. (2008). Applying the Consolidated Standards of Reporting Trials (CONSORT) to studies of mental health provision for juvenile offenders: A research note. Journal of Experimental Criminology, 4, 165–185

[bibr52-1049731513498118] Perry A. E. Weisburd D. Hewitt C. (2010). Are criminologists describing randomized controlled trials in ways that allow us to assess them? Findings from a sample of crime and justice trials. Journal of Experimental Criminology, 6, 245–262

[bibr53-1049731513498118] Pildal J. Hróbjartsson A. Jørgensen K. J. Hilden J. Altman D. G. Gøtzsche P. C. (2007). Impact of allocation concealment on conclusions drawn from meta-analyses of randomized trials. International Journal of Epidemiology, 36, 847–857 17517809 10.1093/ije/dym087

[bibr54-1049731513498118] Prescott R. J. Counsell C. E. Gillespie W. J. Grant A. M. Russell I. T. Kiauka S.…Russel D (1999). Factors that limit the quality, number and progress of randomised controlled trials. Health Technology Assessment, 3, 1–143 10683591

[bibr55-1049731513498118] Schulz K. F. Altman D. G. Moher D. , for the CONSORT Group (2010). CONSORT 2010 statement: Updated guidelines for reporting parallel group randomised trials. British Medical Journal, 340, 698–702

[bibr56-1049731513498118] Schulz K. F. Chalmers I. Hayes R. J. Altman D. G. (1995). Allocation concealment in randomised trials: Defending against deciphering. Lancet, 359, 614–617 11867132 10.1016/S0140-6736(02)07750-4

[bibr57-1049731513498118] Semaan S. Kay L. Strouse D. Sogolow E. Mullen P. D. Neumann M. S.…Des Jarlais D. C (2002). A profile of U.S.-based trials of behavioral and social interventions for HIV risk reduction. Journal of Acquired Immune Deficiency Syndromes, 30, S30–S50 12107358

[bibr58-1049731513498118] Shepperd S. Lewin S. Straus S. Clarke M. Eccles M. P. Fitzpatrick R.…Sheikh A. (2009). Can we systematically review studies that evaluate complex interventions? PLoS Medicine, 6, 31000086 10.1371/journal.pmed.1000086 PMC2717209 19668360

[bibr59-1049731513498118] Stinson J. N. McGrath P. J. Yamada J. T. (2003). Clinical trials in the journal of pediatric psychology: Applying the CONSORT statement. Journal of Pediatric Psychology, 28, 159–167 12654939 10.1093/jpepsy/jsg001

[bibr60-1049731513498118] Tong A. Sainsbury P. Craig J. (2007). Consolidated criteria for reporting qualitative research (COREQ): A 32-item checklist for interviews and focus groups. International Journal for Quality in Health Care, 19, 349–357 17872937 10.1093/intqhc/mzm042

[bibr61-1049731513498118] Torgerson C. J. Torgerson D. J. Birks Y. F. Porthouse J. (2005). A comparison of RCTs in health and education. British Educational Research Journal, 31, 761–785

[bibr62-1049731513498118] Tugwell P. Petticrew M. Kristjansson E. Welch V. Ueffing E. Waters E.…Kelly M. P. (2010). Assessing equity in systematic reviews: Realising the recommendations of the commission on social determinants of health. British Medical Journal, 341, c4739 20837575 10.1136/bmj.c4739

[bibr63-1049731513498118] Turner L. Shamseer L. Altman D. G. Weeks L. Peters J. Kober T.…Moher D. (2012). Consolidated standards of reporting trials (CONSORT) and the completeness of reporting of randomised controlled trials (RCTs) published in medical journals. Cochrane Database of Systematic Reviews, 11, MR000030 23152285 10.1002/14651858.MR000030.pub2 PMC7386818

[bibr64-1049731513498118] von Elm E. Altman D. G. Egger M. Pocock S. J. Gotzsche P. C. Vandenbroucke J. P. (2007). The strengthening the reporting of observational studies in epidemiology (STROBE) statement: Guidelines for reporting observational studies. Annals of Internal Medicine, 147, 573–577 17938396 10.7326/0003-4819-147-8-200710160-00010

[bibr65-1049731513498118] Wang S. Moss J. R. Hiller J. E. (2006). Applicability and transferability of interventions in evidence-based public health. Health Promotion International, 21, 76–83 16249192 10.1093/heapro/dai025

[bibr66-1049731513498118] Welch V. Petticrew M. Tugwell P. Moher D. O’Neill J. Waters E., …the PRISMA-Equity Bellagio group. (2012). PRISMA-Equity 2012 extension: Reporting guidelines for systematic reviews with a focus on health equity. PLoS Medicine, 9, e1001333 10.1371/journal.pmed.1001333 PMC3484052 23222917

[bibr67-1049731513498118] Zwarenstein M. Treweek S. Gagnier J. J. Altman D. G. Tunis S. Haynes B .,…for the CONSORT and Pragmatic Trials in Healthcare (Practihc) group. (2008). Improving the reporting of pragmatic trials: An extension of the CONSORT statement. British Medical Journal, 337, a2390 19001484 10.1136/bmj.a2390 PMC3266844

